# Assessing the sequencing success and analytical specificity of a targeted amplicon deep sequencing workflow for genotyping the foodborne parasite *Cyclospora*

**DOI:** 10.1128/jcm.01811-24

**Published:** 2025-05-14

**Authors:** Anna C. Peterson, David Jacobson, Travis Richins, Joel Barratt, Yvonne Qvarnstrom

**Affiliations:** 1 Centers for Disease Control and Prevention, National Center for Emerging and Zoonotic Infectious Diseases, Division of Parasitic Diseases and Malaria, Laboratory Science and Diagnostics Branch 1242, Atlanta, Georgia, USA; Mayo Clinic Minnesota, Rochester, Minnesota, USA

**Keywords:** molecular epidemiology, foodborne illness, cyclosporiasis, parasitology

## Abstract

Epidemiological investigations of the foodborne parasitic illness cyclosporiasis can be aided by molecular techniques that enable the identification of genetically related clusters of *Cyclospora* isolates. At the Centers for Disease Control and Prevention (CDC), routine *Cyclospora* genotyping for the purpose of informing epidemiological outbreak investigations has occurred since 2018 using clinical stool specimens from case patients diagnosed with cyclosporiasis. This approach involves targeted amplicon deep sequencing of eight genotyping markers, followed by bioinformatic processing through a custom clustering algorithm. However, not all stool specimens submitted to the CDC for genotyping successfully amplify for at least five of the eight genotyping markers, the minimum required to be bioinformatically processed through the clustering algorithm. In this study, we utilized information from clinical stool specimens sent to the CDC from the years 2019 to 2023 to assess if the type of preservative, the age of the specimen, or the method used to diagnose the patient influenced the probability of successfully genotyping parasites from a fecal specimen. Additionally, we assessed the analytical specificity of the *Cyclospora* genotyping workflow by analyzing samples positive for other intestinal parasites, including closely related non-human infecting *Cyclospora* species and other coccidia. We found that stool specimens stored in preservatives had a greater likelihood of sequencing success over time relative to specimens without preservatives or those stored in non-nutritive transport media. Additionally, stool specimens from case patients diagnosed via microscopy-based methods were more likely to yield DNA of sufficient quality and quantity for genotyping compared to PCR or multiplex panels. Lastly, we determined that the genotyping workflow has an analytical specificity of 100%, as no non-human-infecting *Cyclospora* or other parasites yielded sequence data at >1 of the genotyping markers. This knowledge will help strengthen the quality of *Cyclospora* genotyping data produced in the future, improving the utility of this data for supporting epidemiological investigations.

**IMPORTANCE:**

Determining the genetic relatedness among parasites causing foodborne illness, such as *Cyclospora*, is a valuable tool to complement outbreak investigations. However, this molecular genotyping approach is limited by the quality and quantity of genetic data obtained from the samples being investigated. In this study, we demonstrate that the storage conditions of clinical stool specimens are correlated to the quality of sequence data produced for *Cyclospora* genotyping. Our insights can be used to guide storage recommendations for stool specimens, which can improve the quality of foodborne illness outbreak investigations conducted in the future. Additionally, we showed that the current *Cyclospora* genotyping tool used by the Centers for Disease Control (CDC) is highly specific to human-infecting *Cyclospora* parasites; this valuable information indicates that the CDC’s *Cyclospora* investigations are not negatively impacted by false-positive detections.

## INTRODUCTION

Cyclosporiasis is a foodborne gastrointestinal illness caused by human-infecting apicomplexan parasites in the genus *Cyclospora* (1, 2) and is notifiable in the United States (US). Infection occurs via ingestion of the sporulated oocyst stage of the parasite, with human fecal-contaminated produce or water being the most common source (3). The unsporulated stage of the parasite is passed in feces, where it must sporulate at optimal environmental conditions for 1–2 weeks before becoming infective (2). In the US, cyclosporiasis cases have been linked to a wide variety of fresh (primarily) imported produce, including raspberries, basil, cilantro, and several types of lettuce and bagged salad mixes (4), though US-grown produce has also been implicated (5, 6). From 2018 to 2023, between 1,000 and 2,000 domestically acquired cases of cyclosporiasis have been reported each year, and several recalls of contaminated fresh produce have been issued during this timeframe (4). Epidemiological investigations are key to identifying sources of infection, though they can be complicated by the fact that it usually takes several weeks from ingestion of the sporulated oocysts to develop symptoms, limiting patient recall of potential food exposures. As such, molecular tools can play a valuable part in complementing epidemiological investigations (7).

In 2018, the Division of Parasitic Diseases and Malaria (DPDM) at the Centers for Disease Control and Prevention (CDC) began regular, near real-time genotyping of stool specimens from case patients diagnosed with cyclosporiasis to complement epidemiological investigations during the period of heightened US cyclosporiasis activity (usually 1 May to 31 August) (7). State public health labs from across the US submit stool specimens from case patients diagnosed with cyclosporiasis directly to the CDC or to partner labs for genotyping using a targeted amplicon deep sequencing approach (8). This approach uses eight genotyping markers (two mitochondrial and six nuclear markers), which are deeply sequenced using Illumina technology (8, 9). The resulting genetic data is then passed through a CDC-developed clustering algorithm (CYCLONE) to assign each specimen to a genetic cluster (8, 10). Temporal information is then used to further refine relationships by assigning specimens to temporal-genetic clusters (11).

The genetic data generated from the eight genotyping markers has proven useful for complementing epidemiological investigations (7, 11, 12). The performance of the CYCLONE algorithm has been assessed by comparisons to epidemiologically defined outbreak clusters and was found to be highly sensitive and specific at assigning genetic links for successfully genotyped *Cyclospora* (both values >95%) (8). However, a specimen must obtain an amplicon for at least five of the eight genotyping markers to be considered successfully genotyped. In the years 2018–2023, we were able to successfully genotype ~80% of specimens, with the remaining ~20% lacking sufficient data for clustering, which is a likely consequence of insufficient DNA quality and/or quantity.

In this study, we aim to identify the specific features of the clinical stool specimen, such as age, fixative/transport media, or the method by which the case-patient was diagnosed, to determine if these factors influence the likelihood of successfully sequencing at least five of the eight genotyping markers. Additionally, the analytical specificity of the genotyping workflow/CYCLONE algorithm has not been previously assessed. Some of our markers, particularly the mitochondrial markers, target highly conserved regions that are expected to amplify DNA from parasites related to human-infecting *Cyclospora* based on primer homology. However, the propensity for our eight primer sets to amplify DNA from off-target pathogens has not been sufficiently explored. By subjecting several closely related parasites to our laboratory and bioinformatics workflows, we assess whether other off-target parasites yield sufficient sequence data to meet or exceed the minimum data requirement of five markers.

## MATERIALS AND METHODS

### Genotyping

Since 2018, stool from case patients diagnosed with cyclosporiasis has been routinely sent to CDC/DPDM from public health labs throughout the US for genotyping. For each stool specimen submitted, we request information regarding the (i) method used to diagnose the patient, (ii) fixative/transport media in which the stool specimen is stored, and (iii) the date of collection of the stool specimen. However, this information was inconsistently provided in 2018, thus, this dataset only consists of information from specimens sent to the CDC during the years 2019–2023.

Upon arrival at the CDC, each specimen is assigned a unique laboratory identifier and processed through the previously described targeted amplicon deep sequencing genotyping workflow (8). Briefly, this workflow consists of a nucleic acid extraction using the UNEX method (13) followed by a clean-up step to remove potential downstream inhibitors present in the stool. To assess the quality and quantity of DNA present, each specimen is tested using a real-time polymerase chain reaction (PCR) assay, which was designed to target the 18S rRNA gene of human-infecting *Cyclospora* (18S PCR) (13). Beginning in 2021, specimens with a Ct value ≥38 were not processed further as prior years’ experience indicated that these specimens were unlikely to yield a genotype (*n* = 3/3 failed to meet genotyping criteria in years 2019 and 2020). All specimens meeting this Ct threshold are then amplified via conventional PCR using a set of six nuclear and two mitochondrial genotyping markers in eight individual reactions (1, 8). Individual amplicons are normalized for concentration, pooled by specimen, and then prepared for sequencing on an Illumina MiSeq instrument using the Illumina DNA Prep library preparation kits (8, 12).

Resultant data are then passed through the CYCLONE Bioinformatic Workflow (12), which consists of quality filter and trimming steps followed by alignment to a set of reference sequences for each marker to identify haplotypes present in each sample. A pairwise distance matrix is calculated to determine the genetic cluster membership of each specimen meeting certain inclusion criteria. While the workflow can account for some missing data by attempting to impute missing distances, reasonable minimum sequence data requirements must be set (10). For our purposes, we require that for a given specimen, at least five of the eight genotyping markers must possess a sequence before clustering is attempted. Specimens that either exceeded the Ct threshold of 38 or had a sequence for <5 genotyping markers were considered a “fail,” while all other specimens were considered a “pass” (14).

### Data analysis

Due to the variety of responses provided by submitting public health laboratories, we collapsed the different diagnostic methods reported into three categories to ensure consistency: microscopy-based diagnoses (*n* = 871, including submissions that listed general microscopy, UV-florescence, ova and parasite, wet mount, and acid fast/modified acid fast as the diagnostic method), non-multiplex PCR-based diagnoses (*n* = 199, including specimens submitted with PCR or quantitative PCR [qPCR] as the diagnostic method), and multiplex GI panel (*n* = 1,671) (BioFire GI Panel). Specimens that had “other” listed as the diagnostic method with no further detail given, had no diagnostic method provided, or the method listed was “unknown” were removed from the analysis (*n* = 340).

Stools were also stored in a wide variety of fixatives/transport media. In order to complete a more robust analysis, we collapsed these into three categories: (i) non-nutritive transport media (*n* = 2,285), which included Cary-Blair, ParaPak and Protocol Culture and Sensitivity, ParaPak Enteric Plus, Fecal Swab, E-swab, Enteric Transport Medium, and specimens listed as stored in “non-nutritive media”; (ii) specimens stored in any type of fixative (*n* = 520), including formalin-containing fixatives (ParaPak 10% neutral buffered formalin, ParaPak SAF), polyvinyl alcohol (PVA) containing fixatives (ParaPak LV PVA, Zinc-PVA and “modified PVA fixative”), and “eco-friendly” fixatives (ProtoFix, ParaPak EcoFix, and TotalFix); and (iii) specimens not stored in any sort of fixative/transport media (“no media,” “raw,” etc., *n* = 121). There were 155 specimens that listed “other” with no additional detail or listed the fixative/transport media as “unknown.” These “other/unknown” specimens were removed from the analysis. We determined the age of each specimen by calculating the number of days between the date of collection (as reported by the submitter) and the date of DNA extraction at CDC.

We used a generalized linear model with a binomial distribution to determine the relationship between the pass/fail (1/0) genotyping status of a specimen and its age, fixative type, and the diagnostic method. We also included the interaction between sample fixative and specimen age in the model, as well as sample fixative and diagnostic method. As a separate analysis, we also assessed whether Ct values from the 18S PCR differed significantly with genotyping success (pass/fail status) with a t-test. As we did not attempt to sequence specimens that had Ct values >38 in some years of the study (2021–2023), we removed all specimens with Ct values >38 from this analysis.

To assess the analytical specificity of the genotyping assay (defined as the ability of the assay to detect only the intended analyte without cross-reacting with other substances or genetically or biologically similar parasite species), we processed 39 stool specimens that had been confirmed positive for parasites closely related to human-infecting *Cyclospora* using a combination of microscopy and PCR-based assays in clinical diagnostic testing or in previous research studies (i.e., specificity control samples). These same specimens were also previously used to calculate the specificity of other assays used to detect human-infecting *Cyclospora* (15). The specificity control samples included non-human primate infecting *Cyclospora*: *C. papionis* (*n* = 1), *C. colobi* (*n* = 3), and *C. cercopitheci* (*n* = 1), as well as *Cystoisospora belli* (human, *n* = 8), *Giardia duodenalis* (human, *n* = 6), *Cryptosporidium* spp. (human, *n* = 8), *Eimeria* spp. (chicken, *n* = 4), *Dientamoeba fragilis* (human, *n* = 1), *Entamoeba dispar* (human, *n* = 3), *Entamoeba coli* (human, *n* = 1), *Entamoeba hartmanni* (human, *n* = 1), *Entamoeba histolytica* (human, *n* = 1), and another *Entamoeba* sp. (human, *n* = 1) (Table 1). We attempted to run eight replicates of each parasite species or genus, when possible, though we were limited by the availability of material. We opted to include parasites from non-human stools in this analysis to obtain a more accurate analytical specificity of the *Cyclospora* workflow (as the parasite species most closely related to human-infecting *Cyclospora* are non-human parasites) and because there is interest in using this assay with non-clinical specimens (e.g., environmental samples) (6). All specificity control samples were processed and sequenced alongside a negative control specimen, which was DNA extracted from donated stool from a healthy infant (*n* = 3), as well as a positive control specimen (DNA extracted from a stool specimen from a case patient diagnosed with cyclosporiasis and which previously generated all eight markers with the genotyping panel) (*n* = 3). Aliquots from the negative stool were tested with a BioFire GI panel and found to be negative for all pathogens targeted by that panel (16). We analyzed all parasite DNA and negative controls with the *Cyclospora* 18S PCR assay. Regardless of the resulting Ct value, we attempted to sequence all the specificity control samples using the *Cyclospora* genotyping laboratory workflow and the CYCLONE bioinformatic pipeline. We used the following equation to calculate analytical specificity: [True negatives/(True negatives + False positives)] × 100%, where true negatives were defined as specificity control samples that did not pass the workflow inclusion criteria (had sequence data for <5 genotyping markers). False positives were defined as specificity control samples that did pass the workflow inclusion criteria.

**TABLE 1:** Details of specimens included in *Cyclospora* genotyping specificity panel

Organism	Number tested	# Positive qPCR^*a*^	Ct value	# Successfully genotyped	# w/ Haplotypes detected
*Cystoisospora belli*	8	8	38.1, 34.5, 34.1, 33.7, 30.1, 36.0, 33.0, 32.9	0	0
*Giardia* spp.	6	0	N/A	0	0
*Entamoeba* spp.	7	0	N/A	0	0
*Cryptosporidium* spp.	8	1	33.2	0	0
*Dientamoeba fragilis*	1	0	N/A	0	0
*Eimeria* spp.	4	0	N/A	0	2^*b*^
Non-human *Cyclospora*	5	0	N/A	0	2^*b*^

^*a*^ Using the real-time PCR targeting the 18S rRNA gene.

^*b*^ All haplotypes detected were in the mitochondrial MSR marker.

^*c*^ N/A, No specimens generated Ct values with the real-time PCR assay.

## RESULTS

### Genotyping

**Fig 1:**
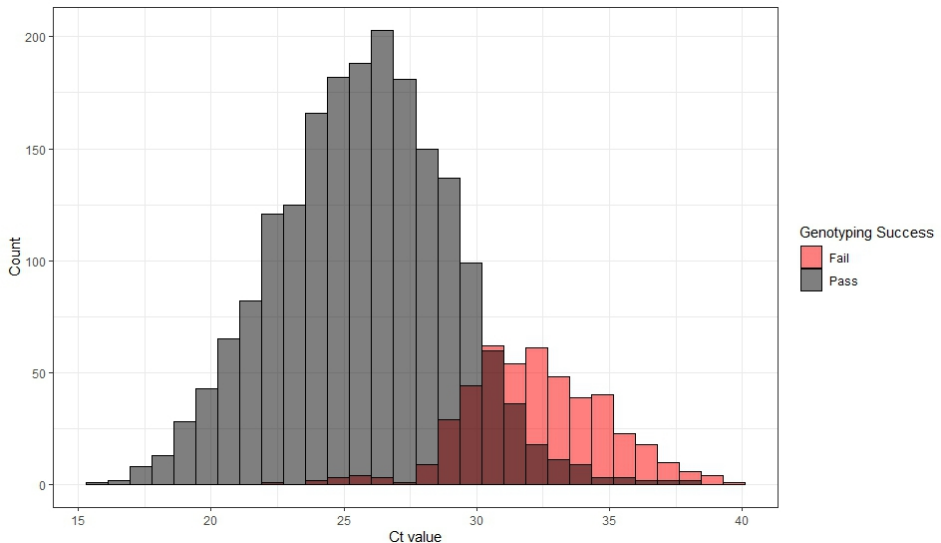
Histogram of the number of specimens (count) by their Ct value from the screening 18S PCR performed at CDC, color coded by if the specimens sequenced at ≥5 markers (pass, in gray) or <5 markers (fail, in red). Above a Ct value of ~33, more specimens tend to fail than pass our genotyping inclusion criteria. This figure includes all specimens received in years 2019–2023 with a Ct value; however, we did not attempt to sequence specimens with Ct values >38 in the years 2021–2023, and those specimens are also represented in red (fail). We also did not attempt to sequence specimens with no Ct value (not represented in the figure). The shaded overlapping bars represent the Ct values at which some specimens passed genotyping criteria, while others failed genotyping criteria. Note, the pass and fail bars are not stacked, thus in each column the shorter shaded bar should be interpreted as the count of the lower category and the higher bar is the count of the higher category (e.g., at the 29–30 Ct bar, 48 samples failed genotyping and 100 passed genotyping).

**Fig 2:**
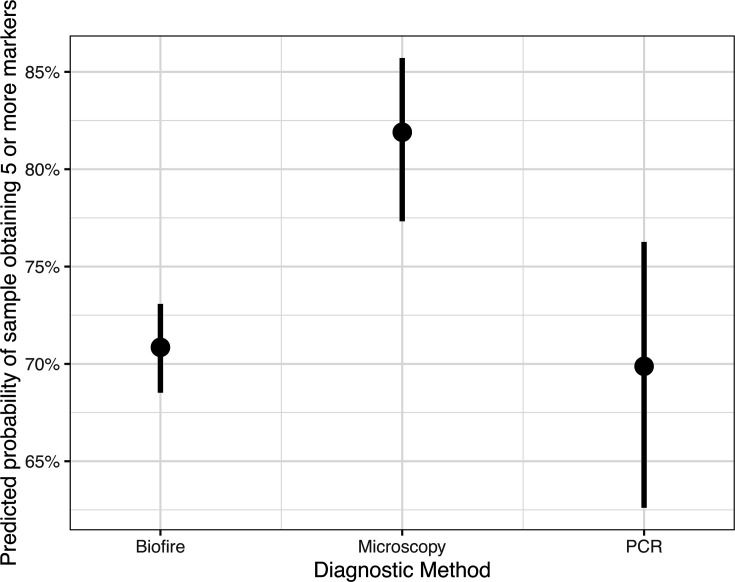
Predicted probability of a sample obtaining ≥5 markers (passing genotyping criteria) by the method used to diagnose the case-patient. Whiskers represent standard error. Specimens from patients diagnosed with microscopy-based methods were significantly more likely to pass genotyping than those from patients diagnosed by either BioFire or PCR. There was not a significant relationship with the fixative type.

**Fig 3:**
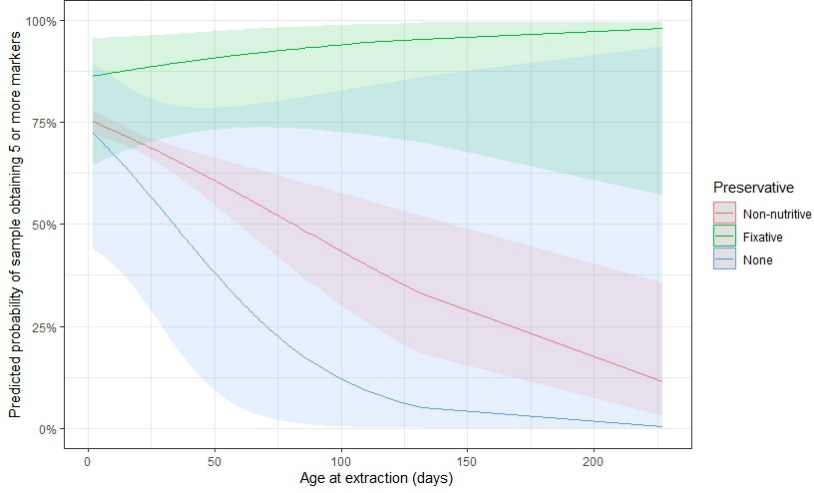
Predicted probability of a specimen passing genotyping by specimen age (in days), among the different categories of preservative/transport media. We were less likely to obtain ≥5 markers from specimens in non-nutritive media and those stored in no type of media/fixative as their age increased relative to specimens in some sort of fixative, for which there was no clear downward trend.

The Ct value of a specimen with 18S PCR was significantly lower for specimens that passed genotyping (mean Ct = 25.8) than those that did not pass genotyping (mean = 32.2) (t = 49.5, d.f. = 1,014.8, *P* < 0.001) (Fig. 1) when considering only specimens with Ct values <38. Of those specimens with Ct values ≥38 for which we attempted sequencing (*n* = 15), only one was successfully genotyped. Specimen age, diagnostic method, and the interaction between fixatives and the age of extraction were all significantly related to the likelihood of a specimen successfully genotyping (all *P*-value < 0.05). Post hoc analysis using Tukey contrasts for multiple comparisons found that specimens from patients diagnosed with microscopy-based methods were more likely to pass genotyping than those from patients diagnosed by either BioFire (coefficient = 0.62, standard error = 0.15, *P*-value < 0.001) or PCR-based methods (coef. = 0.67, SE = 0.22, *P*-value < 0.01) (Fig. 2), though there was not a significant relationship with the fixative type (all *P* > 0.05). The age of a specimen at extraction (mean = 22.3 days, median = 18 days, range 1–227 days) was negatively related to the likelihood of a specimen passing genotyping (coef. = −0.01, SE = 0.004, *P* < 0.001), though there was a significant interaction with the fixative type (*P* = 0.011) (Fig. S1). Specimens in non-nutritive media and those stored in no type of media/fixative were less likely to pass genotyping as their age increased relative to those specimens in some sort of fixative, for which there was no clear downward trend (Fig. 3).

### Specificity

We attempted to sequence all 39 non-*Cyclospora* parasite specimens in our specificity panel using all eight primer sets for the eight genotyping markers. However, none of the specimens in the specificity panel met our minimum data requirement of obtaining a sequence for ≥5 of the eight genotyping markers, and thus were unable to be clustered with the genotyping algorithm. As such, we calculated the analytical specificity of the *Cyclospora* genotyping workflow as 100%. However, we were able to obtain sequences of some mitochondrial haplotypes from some specimens in the panel: including two Mitochondrial small ribosomal RNA subunit (MSR) haplotypes that match human-infecting *Cyclospora* in a highly conserved region of the MSR locus of *C. papionis*, and two haplotypes that match human-infecting *Cyclospora* in more highly variable regions of the MSR locus of *C. colobi*. Lastly, we were able to obtain sequence data from a single haplotype in two of the four *Eimeria* specimens in the specificity panel, each with a haplotype in variable regions of the MSR marker that do not match any *Cyclospora* haplotypes observed in human clinical specimens. We did not calculate the diagnostic specificity of this assay. All positive control specimens passed genotyping (*n* = 3), while none of the negative control specimens produced any haplotypes nor generated Ct values with the 18S PCR assay (*n* = 3).

We did detect potential false positives with the 18S PCR assay, as all eight of the *Cystoisospora* specimens produced Ct values with that assay ranging from 30.8 to 38.1. One of the eight *Cryptosporidium* specimens also had a positive detection with the 18S PCR assay (Ct = 33.3).

## DISCUSSION

Robust molecular data are key to supporting epidemiological investigations of cyclosporiasis outbreaks in the United States (11). Increasing the likelihood of sequencing success for stool samples subjected to our *Cyclospora* genotyping workflow can strengthen the quality and quantity of data produced and ultimately better inform these investigations. In this study, we found that stool specimens stored in specific types of fixative had the highest likelihood of successful genotyping over time, relative to specimens stored in non-nutritive media or specimens stored without fixative or transport media. Stools stored in fixatives/preservatives may have reduced bacterial overgrowth, particularly relative to unpreserved stool. Additionally, preservatives may help to retain the overall integrity of the *Cyclospora* oocysts over time, increasing DNA yields during extraction, as has been seen with the cyst-forming parasite *Giardia* (17), for example. CDC attempts to process stool specimens in as close to real-time as possible, with a mean of 3.5 days from receipt to DNA extraction for specimens with collection dates between 1 May and 31 August (median = 3 days, range 0–48 days). However, there is often a delay between specimen collection and the date it is received at CDC if submitting laboratories need to batch specimens prior to sending or if there is a delay between collection and receipt by the submitting laboratory. The results of this study indicate that specimens not stored in fixatives/preservatives are particularly important to process as soon as possible to increase the likelihood of genotyping success. However, there are limitations to the conclusions that can be drawn from these results. A laboratory study to specifically assess the stability of *Cyclospora* oocysts over time in various media is needed to clarify the timeline at which DNA quality and quantity may begin to decline. Furthermore, some fixatives, such as formalin, are known to inhibit downstream molecular processing (18). Unfortunately, we were unable to assess differences in sequencing success among stool stored in different fixatives due to the small number of samples stored in some fixatives, particularly formalin-containing fixatives (*n* = 4 in this study), though all four formalin-stored specimens failed to sequence at >2 markers, failing genotyping. Additionally, “ecofriendly” fixatives are generally preferred over PVA or formalin-containing fixatives due to the reduced potential for downstream inhibition and to decrease laboratory and environmental hazards (19).

We also found that stool from patients diagnosed with cyclosporiasis microscopically is more likely to yield sufficient sequence data for clustering compared to those diagnosed by either the BioFire GI panel or other PCR-based methods, regardless of preservative type. This may be due to differences in the limit of detection of the different diagnostic tests. Prior work has shown that the BioFire GI panel has a very low limit of detection and can detect even a single *Cyclospora* oocyst in the reaction input volume (16). While the limit of detection of microscopy is not well characterized for *Cyclospora*, it is likely higher than that of the BioFire panel as the former relies on observation of the physical oocysts (16). As such, specimens from patients diagnosed by microscopy may have higher overall parasite levels relative to those diagnosed by the BioFire GI panel, as low-level infections may be missed by microscopy. However, further work needs to be done to establish the limit of detection of the different *Cyclospora* diagnostic assays using comparable metrics.

To our knowledge, false positive detection of *Cyclospora* by the BioFire GI panel has not been reported, although false positive detections have been reported for other targets in this multiplex panel (20, 21). Successful sequencing through our workflow requires relatively high DNA quality and quantity, which is indicated by the fact that we observed a steep decline in sequencing success of specimens with qPCR Ct values >30 (Fig. 1). Thus, the discrepancy observed in sequencing success of microscopy-confirmed cases relative to confirmation via the BioFire GI panel is likely a function of differences in the number of *Cyclospo*ra oocysts present in the stool specimens. Furthermore, in 2021, we instituted a Ct threshold to exclude samples prior to sequencing and did not attempt to sequence specimens with Ct values ≥38. However, instituting a lower threshold could conserve time and sequencing reagents, as specimens with Ct values ≥35 passed sequencing only 10% of the time (10/100 samples with Ct ≥35), and represented 3.6% of specimens received (Fig. 1).

Lastly, we found that the *Cyclospora* genotyping assay has 100% specificity as none of the specificity control samples passed the inclusion criteria for clustering (i.e., successful sequence data from ≥5 of the eight genotyping markers). We were somewhat limited in the availability of non-target coccidian parasites to include in the panel, and it is possible that there may be some closely related coccidia that could cross-react with our genotyping assay. However, we did analyze several specimens of closely related primate *Cyclospora*, and while we were able to sequence one marker (MSR) from these parasites, as well as from an *Eimeria* species, neither yielded data for other markers, and so failed the minimum data requirements for clustering. This indicates it is unlikely that any known parasites in human stool would cross-react with our genotyping assay, given that the most closely known species to human-infecting *Cyclospora* (the two primate *Cyclospora* species) only generated sequence data at a single marker.

We were not able to generate sequence data from any of the specificity panel specimens that produced Ct values with the 18S PCR screening assay, and conversely, the *Eimeria* and *Cyclospora* specificity panel specimens from which we did generate some sequences did not produce Ct values with the 18S PCR assay. Given that many of the haplotypes generated from the *Eimeria* and primate-*Cyclospora* specimens did not match haplotypes observed in human-infecting *Cyclospora*, this result is unlikely to be due to low levels of human-infecting *Cyclospora* DNA present in these samples (such as via contamination or co-infection), and more likely a true amplification of the highly conserved regions of the mitochondrial marker of *Eimeria* and primate-*Cyclospora*. The *Cystoisospora* and *Cryptosporidium* specimens were identified in stool specimens via microscopy (acid fast, O&P, and autofluorescence used), and no *Cyclospora* were detected. However, we cannot rule out the potential that these specimens could have had low levels of *Cyclospora* co-infection that were undetected. This may be more likely for the single *Cryptosporidium* specimen that produced a Ct value with the 18S PCR assay, as other studies have found that *Cryptosporidium parvum, C. hominis*, and *C. meleagridis* do not cross-react with the 18S PCR (22). While non-human *Cystoisospora* (Isospora) have not been found to cross-react with the 18S PCR assay (22), under the specific reaction conditions in this study, we found that *C. belli* from human stool specimens consistently cross-reacted with the 18S rRNA target (*n* = 8/8). These same *Cystoisospora* samples were used to test the specificity of a nested real-time qPCR assay developed for cyclosporiasis diagnostics, and no *Cyclospora* was detected in those samples by that assay (15), lending further support that non-*Cyclospora* coccidian parasites can produce false-positive detections of real-time PCR assays using the 18S rRNA target under some conditions.

Overall, our results indicate that the *Cyclospora* genotyping workflow is highly specific for human-infecting *Cyclospora* species, though the marker that targets the mitochondrial junction did produce haplotypes from non-*Cyclospora* parasites. This assay may be useful for follow-up investigations of unknown specimens with positive detections with the 18S PCR assay (6), but all eight markers should be used. Additionally, care should be taken when interpreting results of such efforts, as even clinical human specimens from case patients diagnosed with cyclosporiasis can fail to amplify at the ≥5 targets needed to cluster specimens, especially when Ct values are >35 with the 18S PCR. When considering clinical human specimens, those stored in preservatives are the most likely to yield high-quality data, even over relatively short time periods (<1 year), though preservatives that do not contain PVA or formalin are preferred. This information should be considered when designing studies for which *Cyclospora*-containing specimens would need to be maintained for longer time periods prior to DNA extraction or when considering the archival of stool specimens. Additionally, new multiplex panels for genotyping *Cyclospora* parasites have recently become commercially available (23). Given that these panels also rely on similar DNA extraction, PCR, and sequencing technologies as used in our study, it is likely that similar stool storage conditions will also increase the likelihood of obtaining high-quality sequence data from stool specimens using those assays.

## SUPPLEMENTAL MATERIAL

Figure S1 jcm.01811-24-s0001.docx Histogram of the age at receipt in days (left) and the age of extraction in days (right) of the stool specimens.

ASM does not own the copyrights to Supplemental Material that may be linked to, or accessed through, an article. The authors have granted ASM a non-exclusive, world-wide license to publish the Supplemental Material files. Please contact the corresponding author directly for reuse.

## References

[1] Barratt JLN, Shen J, Houghton K, Richins T, Sapp SGH, Cama V, Arrowood MJ, Straily A, Qvarnstrom Y. 2023. Cyclospora cayetanensis comprises at least 3 species that cause human cyclosporiasis. Parasitology 150:269–285. doi:10.1017/S003118202200172X 36560856 PMC10090632

[2] Mathison BA, Pritt BS. 2021. Cyclosporiasis-updates on clinical presentation, pathology, clinical diagnosis, and treatment. Microorganisms 9:1863. doi:10.3390/microorganisms9091863 34576758 PMC8471761

[3] Almeria S, Cinar HN, Dubey JP. 2019. Cyclospora cayetanensis and cyclosporiasis: an update. Microorganisms 7:317. doi:10.3390/microorganisms7090317 31487898 PMC6780905

[4] CDC. 2024. Multistate foodborne outbreak notices. Available from: https://www.cdc.gov/foodborne-outbreaks/active-investigations/all-foodborne-outbreak-notices.html. Retrieved 20 Sep 2024.

[5] Administration UFaD. 2020. Del Monte Fresh Produce N.A., Inc. Voluntarily Recalls Limited Quantity of Vegetable Trays in A Multistate Outbreak of Cyclospora Illnesses in Select Retailers in Illinois, Indiana, Iowa, Michigan, Minnesota and Wisconsin, Because of Possible Health Risk. Available from: https://www.fda.gov/safety/recalls-market-withdrawals-safety-alerts/del-monte-fresh-produce-na-inc-voluntarily-recalls-limited-quantity-vegetable-trays-multistate. Retrieved 20 Sep 2024.

[6] Kahler AM, Hofstetter J, Arrowood M, Peterson A, Jacobson D, Barratt J, da Silva A, Rodrigues C, Mattioli MC. 2024. Sources and prevalence of Cyclospora cayetanensis in southeastern U.S. growing environments. J Food Prot 87:100309. doi:10.1016/j.jfp.2024.100309 38815808 PMC11288253

[7] Barratt JLN, Park S, Nascimento FS, Hofstetter J, Plucinski M, Casillas S, Bradbury RS, Arrowood MJ, Qvarnstrom Y, Talundzic E. 2019. Genotyping genetically heterogeneous Cyclospora cayetanensis infections to complement epidemiological case linkage. Parasitology 146:1275–1283. doi:10.1017/S0031182019000581 31148531 PMC6699905

[8] Nascimento FS, Barratt J, Houghton K, Plucinski M, Kelley J, Casillas S, Bennett CC, Snider C, Tuladhar R, Zhang J, Clemons B, Madison-Antenucci S, Russell A, Cebelinski E, Haan J, Robinson T, Arrowood MJ, Talundzic E, Bradbury RS, Qvarnstrom Y. 2020. Evaluation of an ensemble-based distance statistic for clustering MLST datasets using epidemiologically defined clusters of cyclosporiasis. Epidemiol Infect 148:e172. doi:10.1017/S0950268820001697 32741426 PMC7439293

[9] Nascimento FS, Barta JR, Whale J, Hofstetter JN, Casillas S, Barratt J, Talundzic E, Arrowood MJ, Qvarnstrom Y. 2019. Mitochondrial junction region as genotyping marker for Cyclospora cayetanensis. Emerg Infect Dis 25:1314–1319. doi:10.3201/eid2507.181447 31211668 PMC6590752

[10] Jacobson D, Zheng Y, Plucinski MM, Qvarnstrom Y, Barratt JLN. 2022. Evaluation of various distance computation methods for construction of haplotype-based phylogenies from large MLST datasets. Mol Phylogenet Evol 177:107608. doi:10.1016/j.ympev.2022.107608 35963590 PMC10127246

[11] Barratt J, Ahart L, Rice M, Houghton K, Richins T, Cama V, Arrowood M, Qvarnstrom Y, Straily A. 2022. Genotyping Cyclospora cayetanensis from multiple outbreak clusters with an emphasis on a:2176–2180. doi:10.1093/infdis/jiab495 PMC9200147 34606577

[12] Barratt J, Houghton K, Richins T, Straily A, Threlkel R, Bera B, Kenneally J, Clemons B, Madison-Antenucci S, Cebelinski E, Whitney BM, Kreil KR, Cama V, Arrowood MJ, Qvarnstrom Y. 2021. Investigation of US Cyclospora cayetanensis outbreaks in 2019 and evaluation of an improved Cyclospora genotyping system against 2019 cyclosporiasis outbreak clusters. Epidemiol Infect 149:e214. doi:10.1017/S0950268821002090 34511150 PMC8506454

[13] Qvarnstrom Y, Benedict T, Marcet PL, Wiegand RE, Herwaldt BL, da Silva AJ. 2018. Molecular detection of Cyclospora cayetanensis in human stool specimens using UNEX-based DNA extraction and real-time PCR. Parasitology 145:865–870. doi:10.1017/S0031182017001925 29113617 PMC5940589

[14] Jacobson DK, Peterson AC, Qvarnstrom Y, Barratt JLN. 2023. Novel insights on the genetic population structure of human-infecting Cyclospora spp. and evidence for rapid subtype selection among isolates from the USA. Curr Res Parasitol Vector Borne Dis 4:100145. doi:10.1016/j.crpvbd.2023.100145 37841306 PMC10569985

[15] Richins T, Houghton K, Barratt J, H Sapp SG, Peterson A, Qvarnstrom Y. 2023. Comparison of two novel one-tube nested real-time qPCR assays to detect human-infecting Cyclospora spp. Microbiol Spectr 11:e0138823. doi:10.1128/spectrum.01388-23 37819113 PMC10715049

[16] Peterson A, Richins T, Houghton K, Mishina M, Sharma S, Sambhara S, Jacobson D, Qvarnstrom Y, Cama V. 2023. The limit of detection of the biofire filmarray gastrointestinal panel for the foodborne parasite Cyclospora cayetanensis. Diagn Microbiol Infect Dis 107:116030. doi:10.1016/j.diagmicrobio.2023.116030 37572510 PMC10530562

[17] Wilke H, Robertson LJ. 2009. Preservation of giardia cysts in stool samples for subsequent PCR analysis. J Microbiol Methods 78:292–296. doi:10.1016/j.mimet.2009.06.018 19576935

[18] Fiallo P, Williams DL, Chan GP, Gillis TP. 1992. Effects of fixation on polymerase chain reaction detection of mycobacterium leprae. J Clin Microbiol 30:3095–3098. doi:10.1128/jcm.30.12.3095-3098.1992 1452690 PMC270594

[19] Pietrzak-Johnston SM, Bishop H, Wahlquist S, Moura H, Da Silva ND, Da Silva SP, Nguyen-Dinh P. 2000. Evaluation of commercially available preservatives for laboratory detection of helminths and protozoa in human fecal specimens. J Clin Microbiol 38:1959–1964. doi:10.1128/JCM.38.5.1959-1964.2000 10790128 PMC86633

[20] Jo SJ, Kang HM, Kim JO, Cho H, Heo W, Yoo IY, Park YJ. 2021. Evaluation of the biofire gastrointestinal panel to detect diarrheal pathogens in pediatric patients. Diagnostics (Basel) 12:34. doi:10.3390/diagnostics12010034 35054200 PMC8774520

[21] Matic N, Lawson T, Young M, Jang W, Bilawka J, Gowland L, Ritchie G, Leung V, Payne M, Stefanovic A, Romney MG, Lowe CF. 2024. Melting curve analysis reveals false-positive norovirus detection in a molecular syndromic panel. J Clin Virol 173:105697. doi:10.1016/j.jcv.2024.105697 38820917

[22] Lalonde L, Oakley J, Fries P. 2022. Verification and use of the US-FDA BAM 19b method for detection of Cyclospora cayetanensis in a survey of fresh produce by CFIA laboratory. Microorganisms 10:559. doi:10.3390/microorganisms10030559 35336134 PMC8954584

[23] Leonard SR, Mammel MK, Almeria S, Gebru ST, Jacobson DK, Peterson AC, Barratt JLN, Musser SM. 2024. Evaluation of the increased genetic resolution and utility for source tracking of a recently developed method for genotyping Cyclospora cayetanensis. Microorganisms 12:848. doi:10.3390/microorganisms12050848 38792677 PMC11124223

