# Supplementary material for: Assessing the sequencing success and analytical specificity of a targeted amplicon deep sequencing workflow for genotyping the foodborne parasite Cyclospora
Source: J Clin Microbiol. 2025 May 14;63(6):e01811-24. doi: 10.1128/jcm.01811-24 (PMC12153321; doi:10.1128/jcm.01811-24)

**Supplemental Figure 1.** Histogram of the age at receipt in days (left) and the age of extraction in days (right) of the stool specimens sent to CDC from 2019-2023 and included in our analysis. Bars are stacked, with dark grey bars representing the number of specimens (count) from which we were able to obtain ≥5 genotyping markers and cluster with our bioinformatic algorithm (pass), and the number of specimens that we were unable to obtain at least 5 markers and thus failed to cluster with our genotyping algorithm represented in light grey.


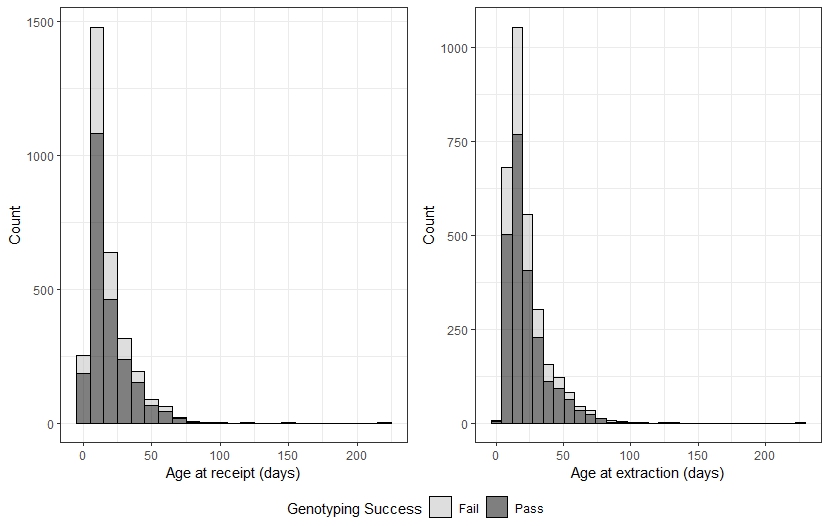

Supplement: Figure S1 — Histogram of the age at receipt in days (left) and the age of extraction in days (right) of the stool specimens. [file jcm.01811-24-s0001.docx]
